# Supplementary figures and images for: Age-related differences in affective behaviors in mice: possible role of prefrontal cortical-hippocampal functional connectivity and metabolomic profiles
Source: Front Aging Neurosci. 2024 Mar 8;16:1356086. doi: 10.3389/fnagi.2024.1356086 (PMC10957556; doi:10.3389/fnagi.2024.1356086)

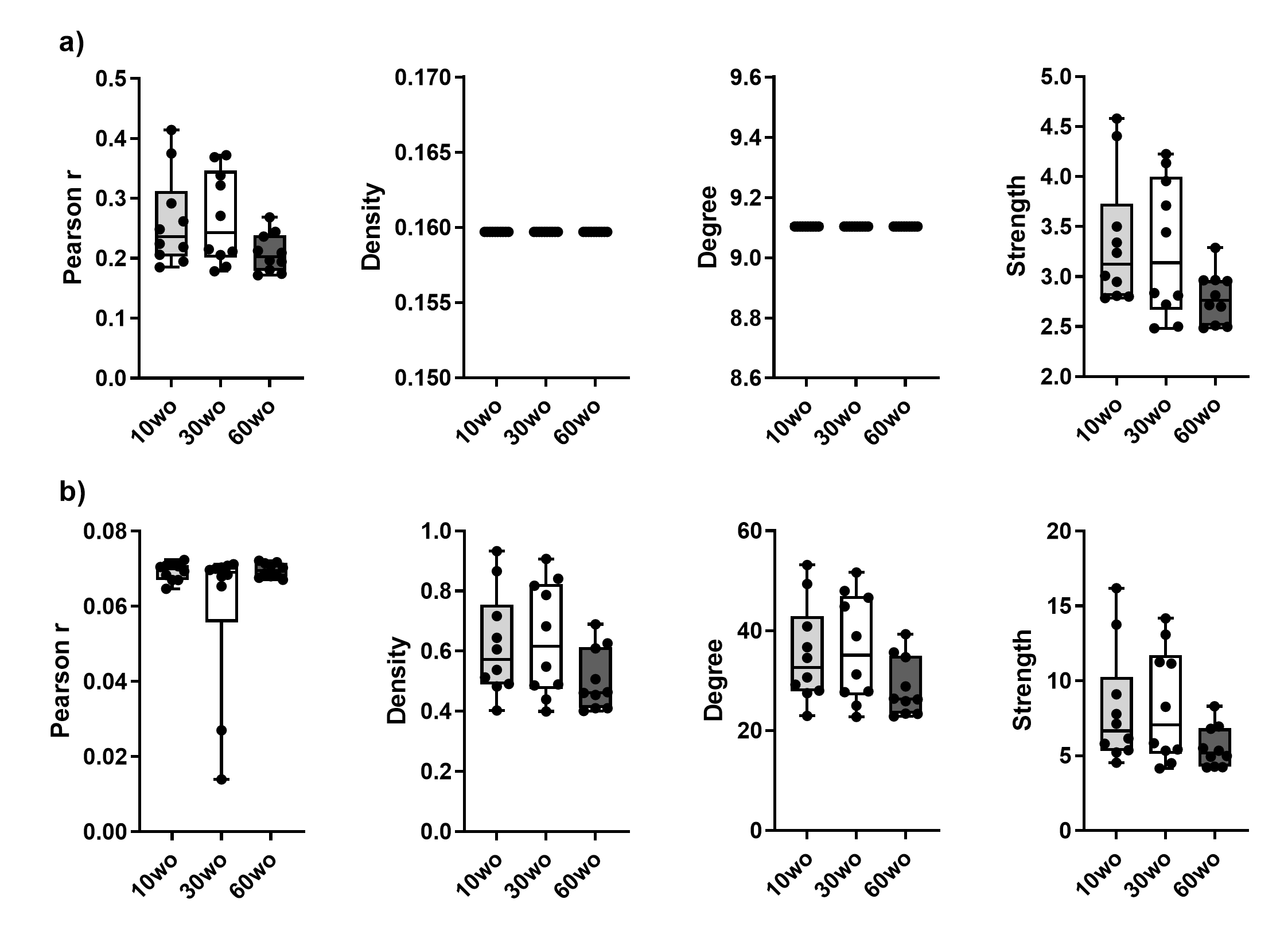

Supplement: Supplementary file 1 [file Image_1.TIF]

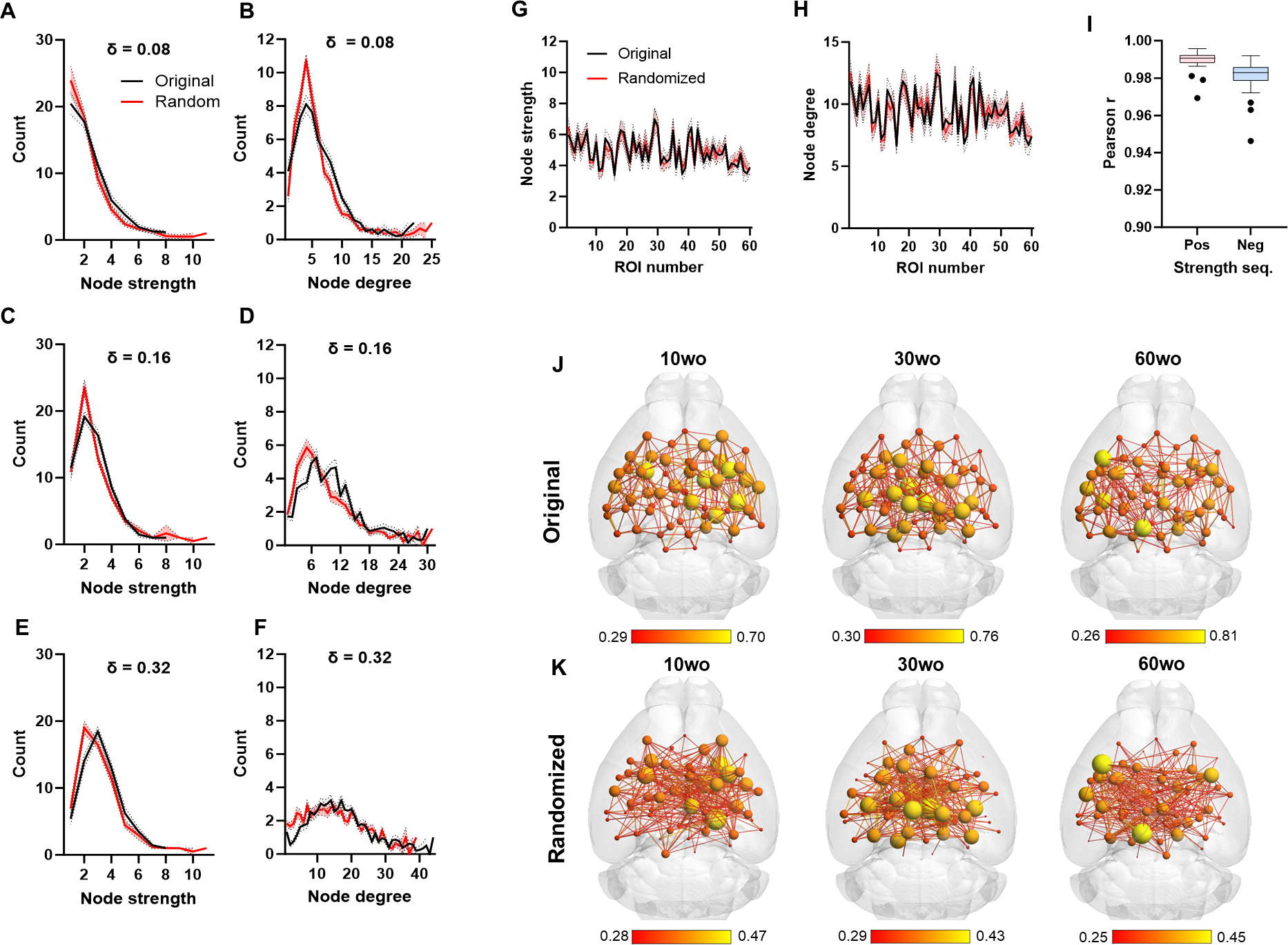

Supplement: Supplementary file 2 [file Image_2.TIF]

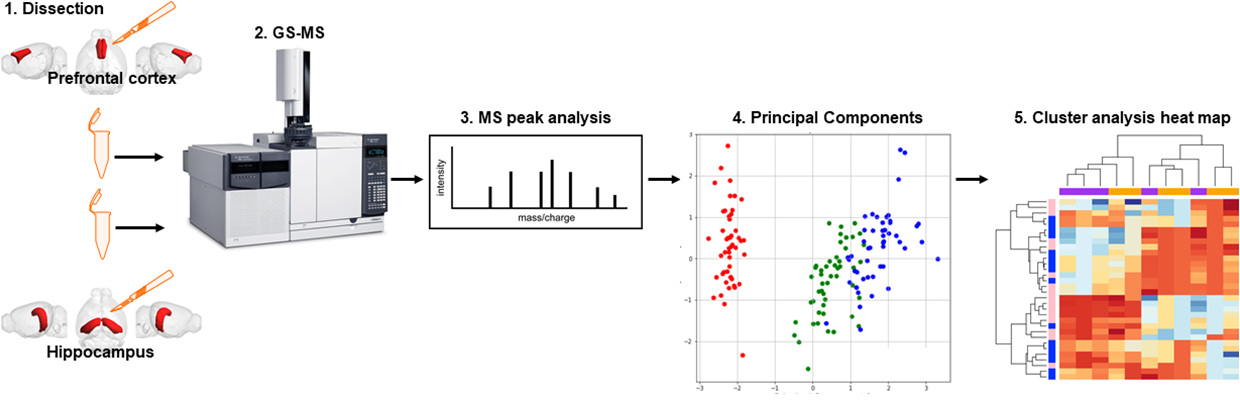

Supplement: Supplementary file 3 [file Image_3.TIF]

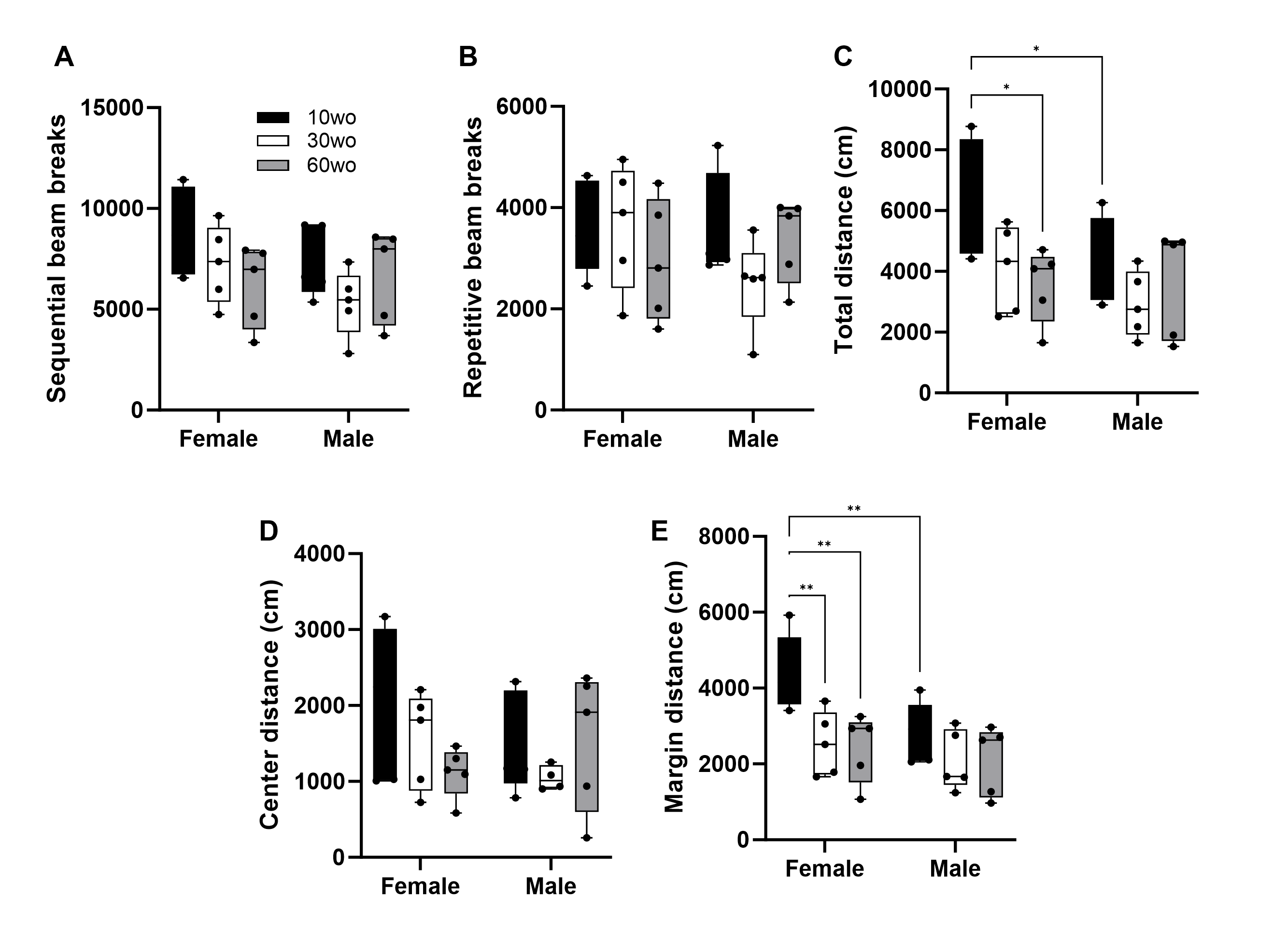

Supplement: Supplementary file 4 [file Image_4.TIF]

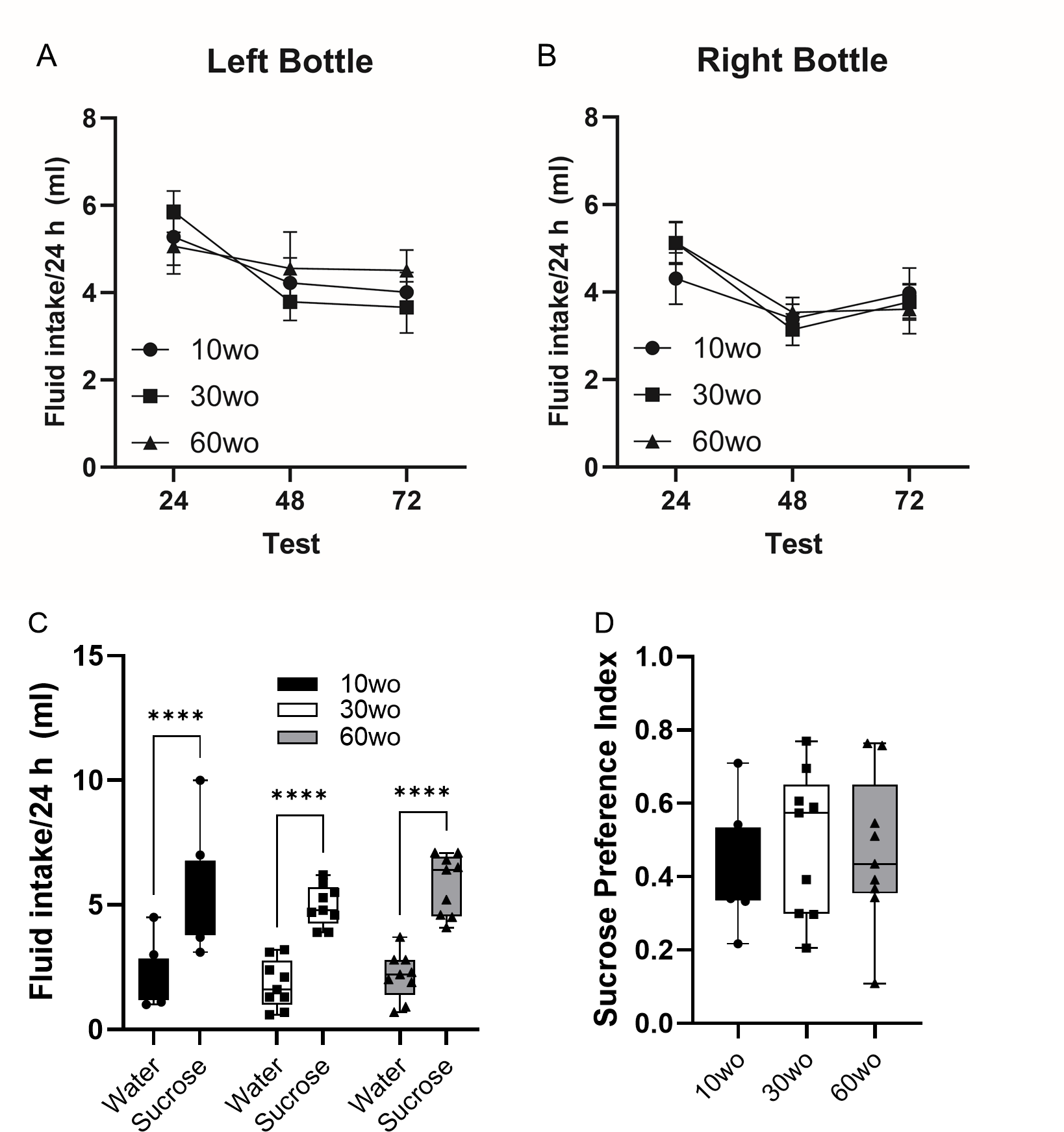

Supplement: Supplementary file 5 [file Image_5.TIF]

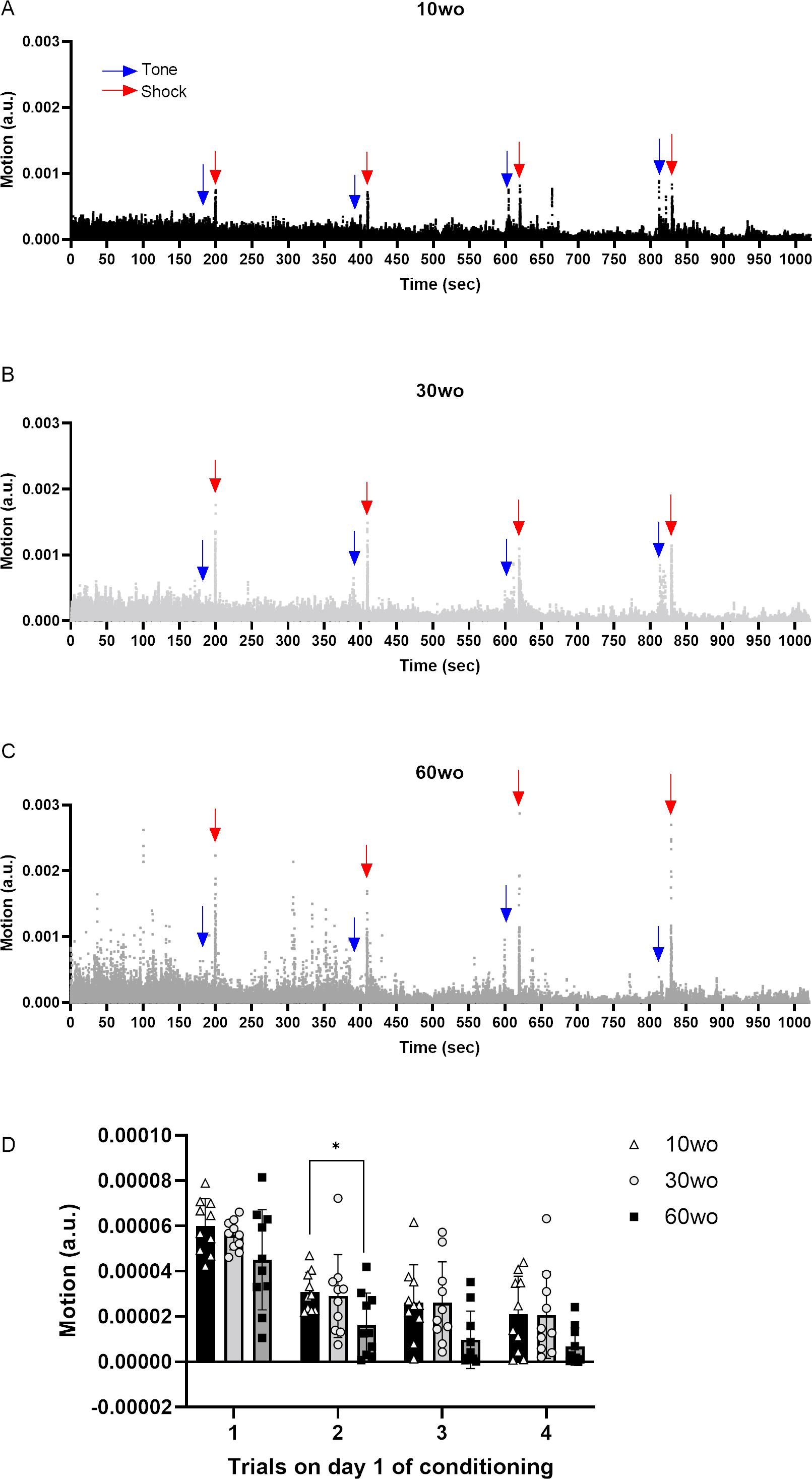

Supplement: Supplementary file 6 [file Image_6.TIF]

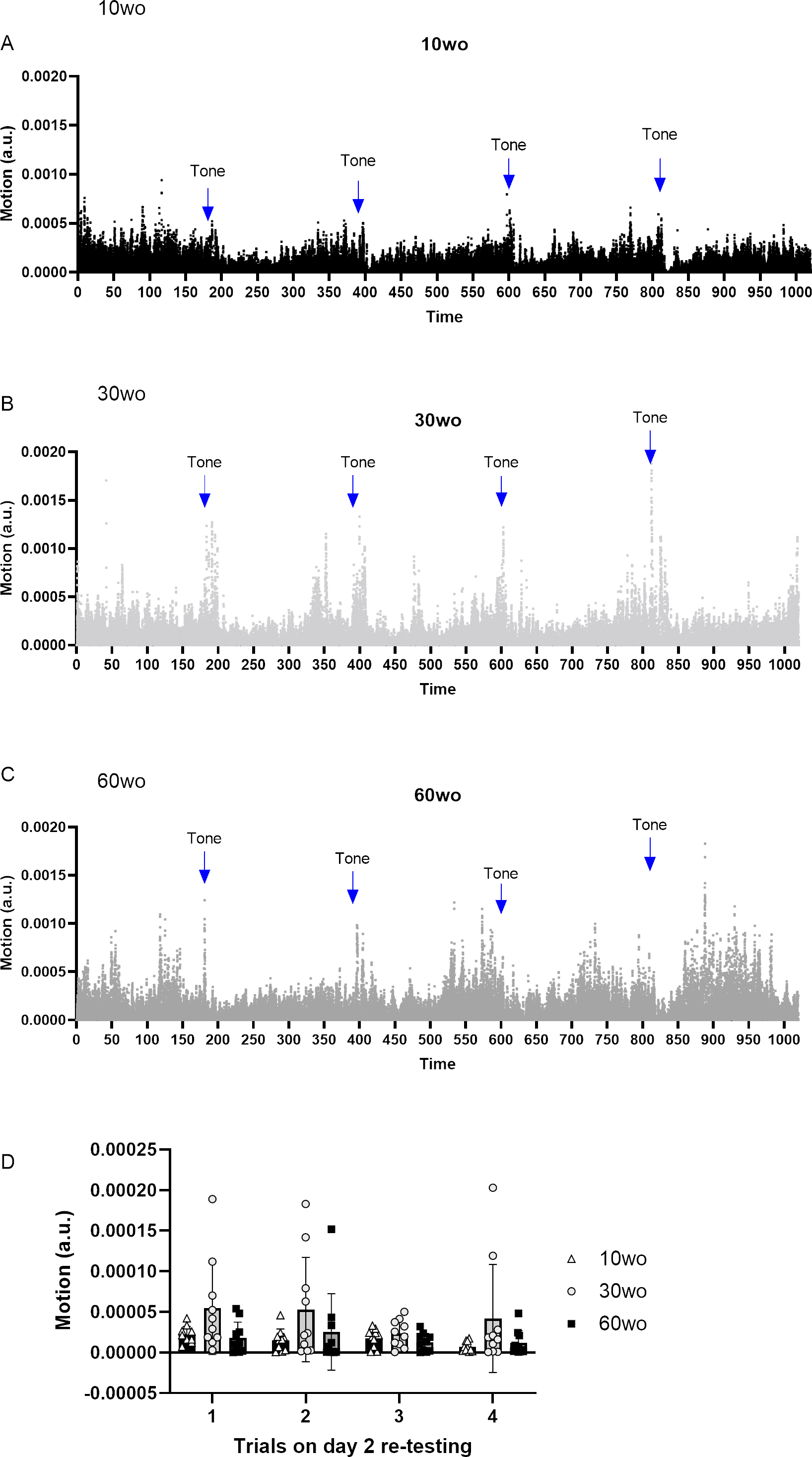

Supplement: Supplementary file 7 [file Image_7.TIF]

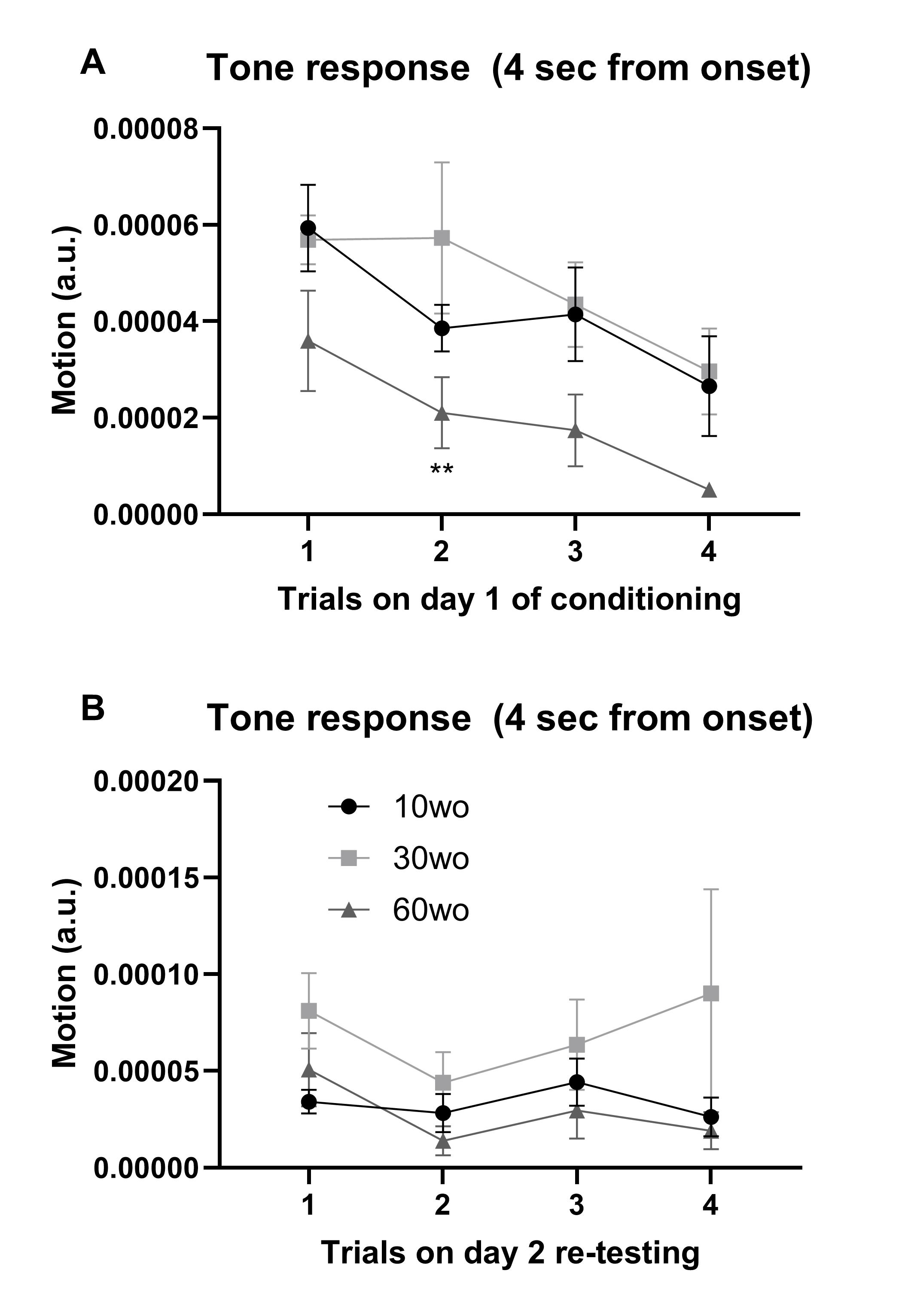

Supplement: Supplementary file 8 [file Image_8.TIF]

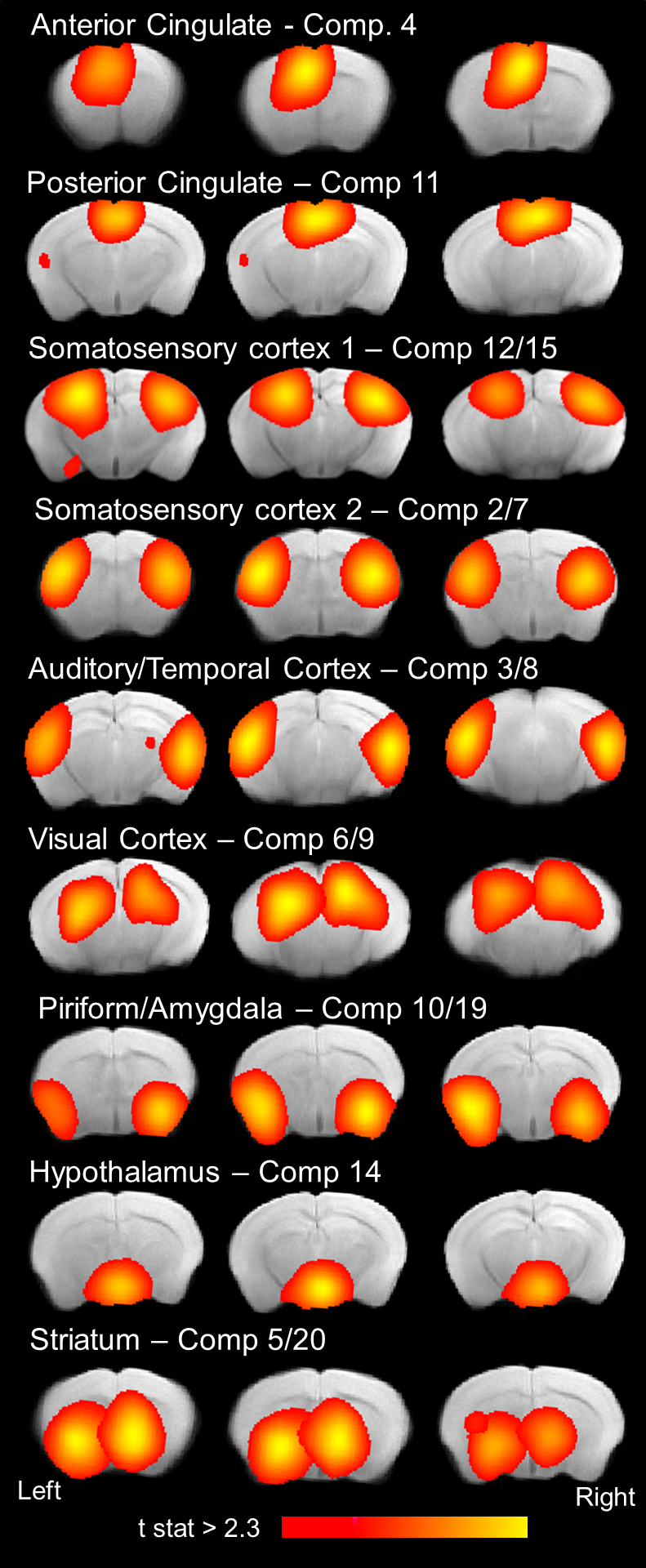

Supplement: Supplementary file 9 [file Image_9.TIF]

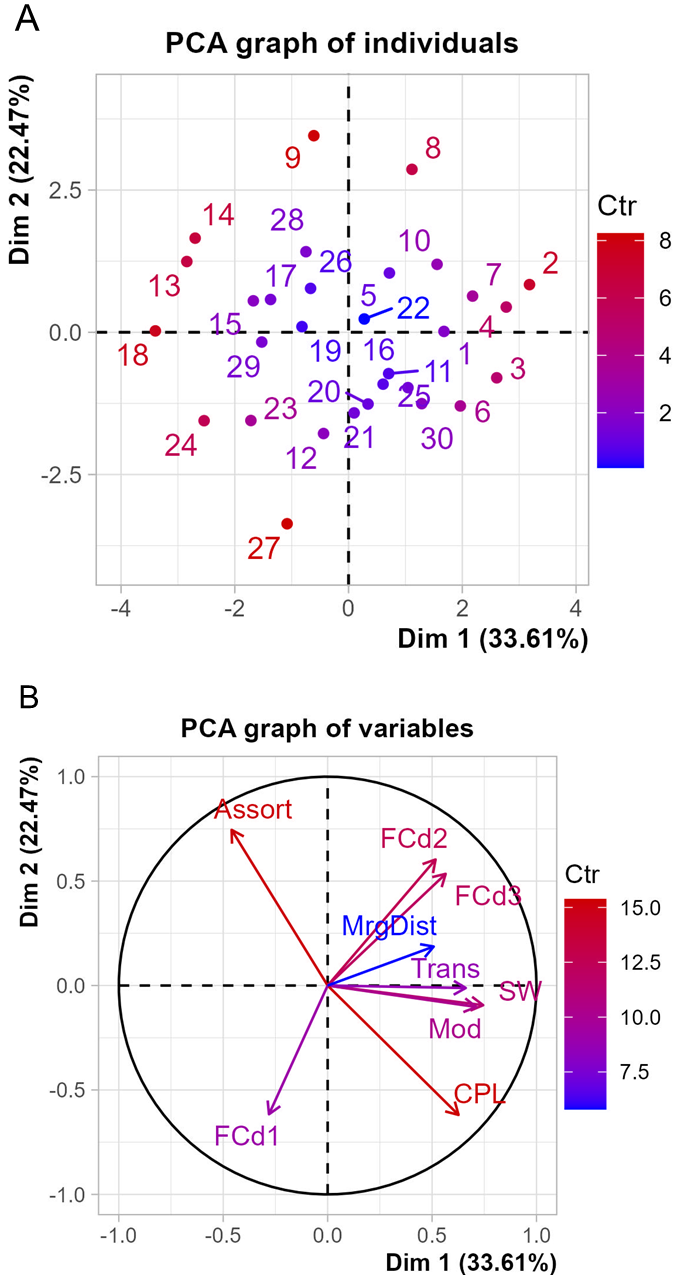

Supplement: Supplementary file 10 [file Image_10.TIF]

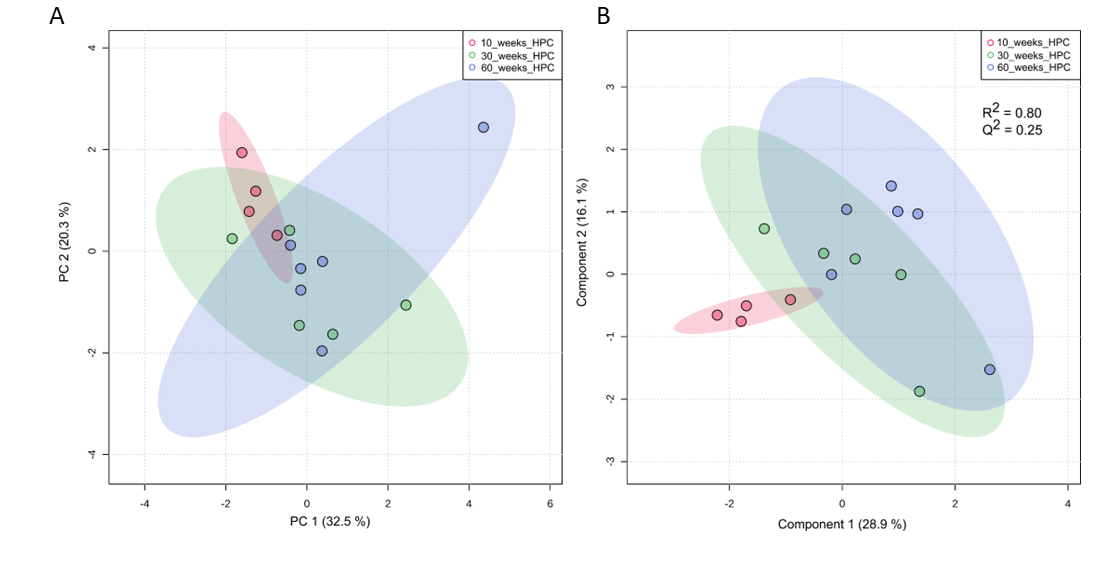

Supplement: Supplementary file 11 [file Image_11.TIF]

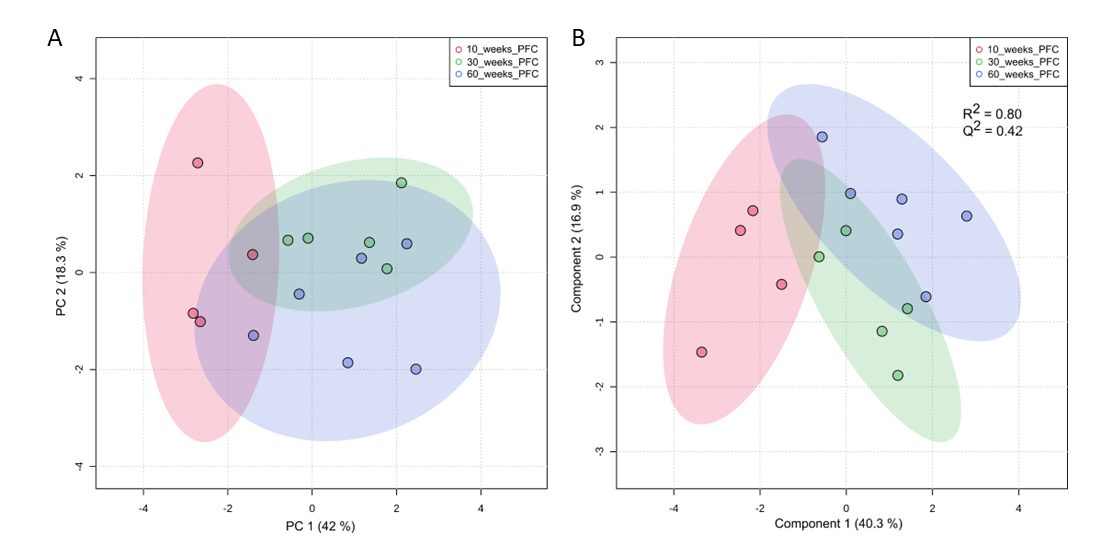

Supplement: Supplementary file 12 [file Image_12.TIF]
